# Bisphenol A: Potential Factor of Miscarriage in Women in the Context of the Phenomenon of Neutrophil Extracellular Traps

**DOI:** 10.1007/s00005-022-00661-w

**Published:** 2022-10-01

**Authors:** Wioleta Justyna Omeljaniuk, Angelika Edyta Charkiewicz, Marzena Garley, Wioletta Ratajczak-Wrona, Jan Czerniecki, Ewa Jabłońska, Marzanna Cechowska-Pasko, Wojciech Miltyk

**Affiliations:** 1 Department of Analysis and Bioanalysis of Medicines, Medical University of Bialystok, Bialystok, Poland; 2 Department of Public Health, Medical University of Bialystok, Bialystok, Poland; 3 Department of Immunology, Medical University of Bialystok, Bialystok, Poland; 4 Biology and Pathology of Human Reproduction, Institute of Animal Reproduction and Food Research, Polish Academy of Sciences in Olsztyn, Olsztyn, Poland; 5 Department of Pharmaceutical Biochemistry, Medical University of Bialystok, Bialystok, Poland

**Keywords:** BPA, Miscarriage, NOX1, NCF2, MCP-1, TNF-α, Anti-PR3, Anti-MPO, NETs

## Abstract

Humans are exposed to a number of environmental pollutants every day. Among them, endocrine disruptors are particularly harmful to human health. Bisphenol A (BPA) is a xenoestrogen that has been shown to disrupt the endocrine system and cause reproductive toxicity. In this study, we aimed to verify the potential relationship between BPA and miscarriage involving the formation of neutrophil extracellular traps (NETs). Blood samples were collected from healthy women and women who had miscarriage in the first trimester of pregnancy. The serum levels of cytoplasmic anti-PR3 antibody and perinuclear anti-MPO antibody were determined using an immunoenzymatic method. The concentrations of key proinflammatory proteins TNF-α and MCP-1, as well as NADPH oxidase subunits NOX1 and NCF2, were also measured in the serum samples. The serum concentration of BPA was determined using gas chromatography. The results showed that the concentrations of BPA were significantly elevated in the serum of women who had miscarriage compared to the control group, with the highest concentration found in the “NETs-positive” group. The levels of MCP-1 and TNF-α were significantly higher in the “NETs-positive” group compared to the “NETs-negative” and control group. The levels of NOX1 and NCF2 were also higher in the “NETs-positive” group compared to the “NETs-negative” group. The study showed that BPA could play a role in the course of miscarriage through the formation of NETs. The results indicate the need to limit the exposure of women planning pregnancy to xenoestrogens, including BPA.

**Supplementary Information:**

The online version contains supplementary material available at 10.1007/s00005-022-00661-w.

## Introduction

Factors that cause the first miscarriage may also contribute to subsequent miscarriages particularly if they are undetermined. The significance of immune dysfunctions in the etiology of miscarriage has been widely investigated in recent years (American College of Obstetricians and Gynecologists’ Committee on Practice Bulletins—Gynecology 2018; Weeks and Gemzell 2006). The placental changes observed in our previous study suggested two possible mechanisms that could contribute to miscarriage: 1) formation of neutrophil extracellular traps (NETs) and 2) disturbance of oxidative–antioxidative balance (Omeljaniuk et al. 2015, 2020).

In an organism, NET formation is a basic nonspecific strategy protecting against pathogens. However, excessive production of NETs or their long-term persistence may disrupt the balance between the formation and clearance of these structures, inducing inflammatory processes, such as autoimmunization (Brinkmann et al. 2004; Gupta et al. 2005; Hahn et al. 2012). NETs are composed of chromatin and neutrophil granule proteins, which include primary (myeloperoxidase, proteinase 3, neutrophil elastase, cathepsin G, defensins), secondary (lactoferrin), and tertiary (gelatinase) ones, as well as histones (H1, H2A, H2B, H3, H4) with the H2A–H2B–DNA complex (Brinkmann et al. 2004; Brinkmann 2018).

To better understand the role of immune mechanisms in the formation of NETs and the processes related to the generation of free radicals during miscarriage, we decided to expand our study by assessing the concentrations of nicotinamide adenine dinucleotide phosphate oxidase 1 (NOX1), which is one of the isoforms of nicotinamide adenine dinucleotide phosphate hydrogen (NADPH), and that of NCF2 (neutrophil cytosolic factor 2, NOXA2), which is an integral protein of the NADPH oxidase (Chocry and Leloup 2020). To determine the intensity of inflammation that promotes the formation and long-term persistence of NETs (Oliveira de Sousa et al. 2021) in the placenta, we estimated the levels of tumor necrosis factor (TNF)-α and monocyte chemoattractant protein (MCP)-1, which are key proinflammatory proteins synthesized by cells, including neutrophils, and also exert an autocrine effect on these cells (Suzuki et al. 2002).

The hypothesis that the loss of pregnancy is associated with immunoreactions similar to those observed following an allograft encouraged us to identify the autoimmune etiology of miscarriage (Tasadduq et al. 2021). As the production of antineutrophil cytoplasmic antibodies (ANCA) may be induced by the release of NETs, we decided to assess the levels of anti-proteinase 3 (anti-PR3) and anti-myeloperoxidase (anti-MPO) antibodies, which are cytoplasmic and perinuclear ANCA, respectively (Fu et al. 2020).

Given the multifactorial origin of miscarriage, it is necessary to identify the potential factors that trigger genetic, immune, endocrine, morphological and anatomical, infectious, and iatrogenic lesions, which can be observed in women with this type of obstetric failure (Zejnullahu et al. 2021). Bisphenol A (BPA), a xenoestrogen, has been gaining increasing interest due to its unfavorable multidirectional effects (Sekizawa 2008). BPA interacts not only with the hormone system but also with the immune system, and the effect of this xenoestrogen may manifest even after years of exposure or as a result of its accumulation in the body. The expanding global presence of BPA in many forms, ranging from food containers to toys, medical devices, and others, may be a secondary cause for disturbances that eventually result in miscarriage in women (Elobeid and Allison 2008; Keri et al. 2007; Kharrazian 2014; Lathi et al. 2014; Richter et al. 2007; Rutkowska and Rachon 2014; Soto et al. 1995).

**Fig. 1:**
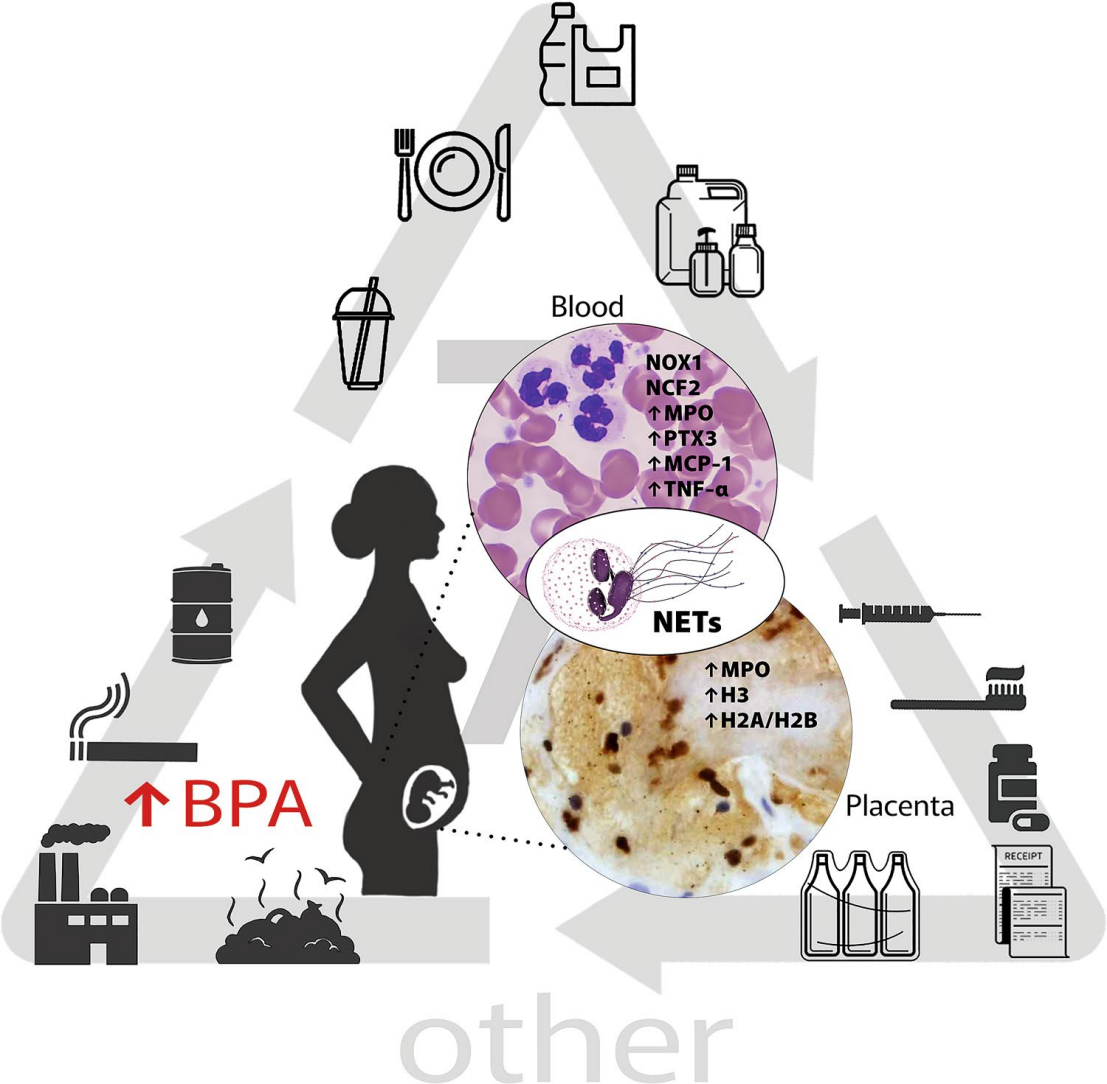
Proposed model of the sources of BPA and its effects at high concentrations in women with miscarriage

We present a model of the sources of BPA and its effects at high concentrations in women with miscarriage in Fig. 1.

## Materials and Methods

### Test and Control Group

The study included a total of 78 females (aged 18–44 years) who miscarried up to 24 weeks of gestation. The patients were hospitalized at the Clinic of Obstetrics and Perinatology of the University Clinical Hospital in Bialystok and at the Maternity Ward with Pregnancy Pathology of the Jędrzej Śniadecki Regional Hospital Complex in Bialystok. These women did not have other diseases. Women diagnosed with antiphospholipid antibody syndrome, vein thrombosis, inflammation, or genetic diseases were excluded. The women in the study group were not receiving any treatment. All the recruited women were from the same geographic region.

The control group comprised ten healthy women (aged 18–32 years) who had a normal course of gestation and had given birth to healthy children. This group was carefully selected, and women with chronic or temporary diseases during gestation were excluded. The characteristics of the women included in the study are presented in Table 1.

**Table 1:** Basic characteristics of the study participants

Women *n* = 88	Number of miscarriages	Week of pregnancy at which miscarriage occured	Week of pregnancy at which blood was collected	Age [years]	BMI [kg/m^2^]	Number of women smoking cigarettes
	Median SD					
“NETs-negative” *n* = 48	1 0.64	8 1.9565	–	31 6.4271	22.465 3.3702	5
“NETs-positive” *n* = 30	1 0.5833	9 2.3116	–	29 5.7517	22.3 4.0343	9
Control group *n* = 10	0 0	–	8.5 2.7162	28.5 2.4967	21.65 2.7165	0

### Materials

The study material was serum samples collected from the test and control groups. For the test group, serum was collected immediately after miscarriage, whereas for the control group, samples were collected during a routine examination in the first trimester of pregnancy. Written consent was obtained from both groups of women before sample collection.

Serum samples were collected as a part of the project entitled “Influence of diet and tobacco smoking on the content of zinc, selenium, copper, manganese and antioxidative status in women with miscarriage”, which was funded by a supervisor research grant of the Ministry of Science and Higher Education (application number: N N405 625,538). Samples were collected at the same time from both test and control groups during 2007–2011. The samples were stored at – 80 °C in accordance with the principles of Good Laboratory Practice. All samples were stored for the same duration under identical conditions.

Approval for the extended analysis of the study material was obtained from the Bioethics Committee of the Medical University of Bialystok (Resolution No. R-I-002/404/2019 on 26 September 2019 and APK.002.423.2020 on 17 December 2020).

### ELISA Tests

The levels of anti-PR3-hn-hr and anti-MPO in the serum samples collected from the examined women were measured using enzyme-linked immunosorbent assay (ELISA) kits obtained from Euroimmun Medizinische Labordiagnostica AG (Lübeck, Germany).

The concentrations of NOX1 and NCF2 in the serum were measured using ELISA kits obtained from Cloud-Clone Corp. (Katy, TX, USA).

The levels of MCP-1 and TNF-α in the serum were measured using ELISA kits obtained from Sigma-Aldrich™ (St. Louis, MO, USA).

**Fig. 2:**
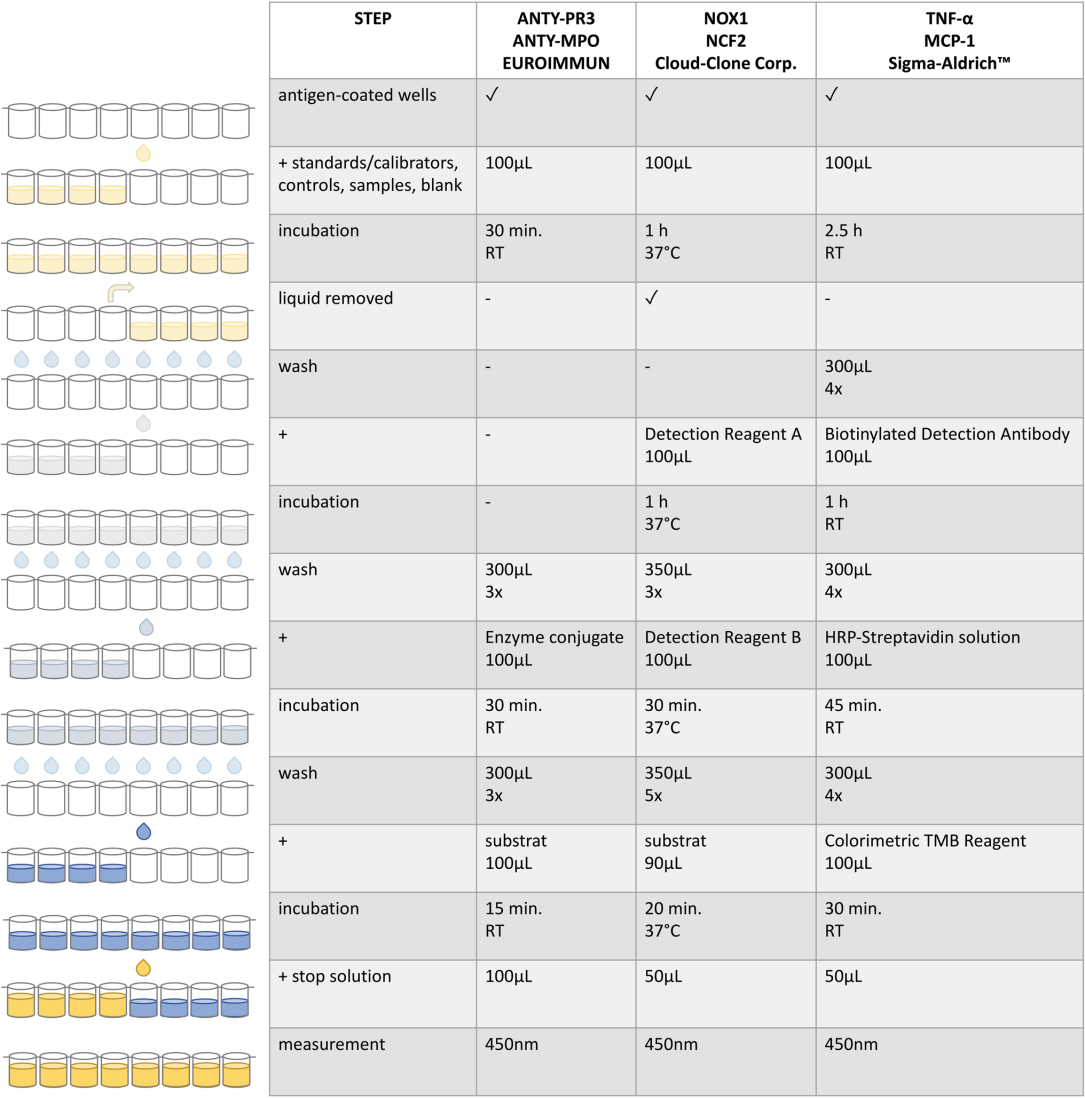
Procedures used for determining the concentrations of the analyzed antibodies and protein

The procedures used for determining the concentrations of the analyzed antibodies and proteins are presented in Fig. 2.

### GC–Mass Spectrometry Analysis

The level of BPA in the serum samples was determined using gas chromatography (GC).

Briefly, serum samples were thawed at room temperature, vortexed, and transferred to polypropylene tubes strengthened with BPA16d (Sigma-Aldrich™, Steinheim, Germany). Then, the samples were treated with chloroform (J. T. Baker, Gliwice, Poland) and acetonitrile (Sigma-Aldrich™, Steinheim, Germany) and sonicated for 1 min in an ice bath. Subsequently, the samples were centrifuged at 5000 rpm and 4 °C, and the resulting aqueous and organic phases were separated. The organic phase was transferred to glass vials and evaporated in a vacuum concentrator (Eppendorf). Finally, pyridine and BSTFA (N,O-bis(trimethylsilyl)trifluoroacetamide; Sigma-Aldrich™, Steinheim, Germany) were added to the vials containing sample for derivatization, before GC.

GC–mass spectrometry analysis was performed using the Pegasus 4DGC × GC–TOFMS system (LECO Corporation, St. Joseph, MO, USA). Briefly, the samples were separated on a 30 m × 0.25 mm, 25-μm thick-film capillary column (SGE Analytical Science Ringwood, Australia). The column temperature was increased from 130 °C to 300 °C at a rate of 10 °C/min. The mobile phase used was ultrapure helium, and its flow rate was maintained at 1.0 mL/min, with an in-built filter for oxygen and humidity. A derivatized sample of 1-μL volume was injected in the splitless mode. The temperature of the injector and that of the mass spectrometry ion source were maintained at 250 °C. The solvent delay was set at 480 s, and the extraction coefficient at 10 spectra/s.

ChromaTOF version 4.51.6.0 software was used to control the device as well as to obtain and assess the data. The concentration of BPA was calculated using the calibration curve for a concentration range 0–1000 ng/mL.

All analyses were performed using BPA-free disposable equipment (tubes, tips); routine diagnostic tubes that were not BPA-free were used only for blood collection. Identical test tubes were used for sample collection for all women.

### Statistical Analysis

The obtained results were analyzed using STATISTICA version 13.3 program (StatSoft, Inc., Tulsa, OK, USA). Quantitative data were presented as median with minimum (min.) and maximum (max.) values or with standard deviation. Data distribution was verified by the Shapiro–Wilk test. Comparisons between groups were performed using Mann–Whitney *U* test or Kruskal–Wallis analysis of variance. Comparison of enumeration data was performed using the chi-square test. Correlations between variables were tested using nonparametric Spearman’s rank correlation coefficient. *p* values less than 0.05 were considered statistically significant.

## Results

The findings of our previous study encouraged us to analyze the potential causes of miscarriage in two different groups of women who had this obstetric condition (Omeljaniuk et al. 2020). The first group included women with miscarriage who did not have NET structures in the placental tissue and were referred to as “NETs-negative” (*n* = 48). The second group included women with miscarriage who had NETs identified within the placental tissue and were referred to as “NETs-positive” (*n* = 30). Because the experimental hypotheses and study results were a continuation of our previous project, data analysis was also performed based on this division.

### Anti-PR3 and Anti-MPO Level Assessment

The assessment of the levels of anti-PR3 and anti-MPO antibodies in the serum of the tested women indicated negative results (a value ≥ 20 relative units indicated a positive result), as shown in Table 2.

**Table 2:** Summary of the results

Women *n* = 88	Anti-PR3 Antibody (cANCA) [RU/mL]	Anti-MPO Antibody (pANCA) [RU/mL]	NOX1 [ng/mL]	NCF2 [ng/mL]	MCP-1 [pg/mL]	TNF-α [pg/mL]	BPA [ng/mL]
	Median Min–Max						
“NETs-negative” *n* = 48	1.2720 0.509–4.137	1.3130 0.196–8.036	8.7630* 5.157–11.979	0.5900* 0.122–1.666	59.4060 33.173–80.224	307.5325 135.323–553.392	27.2700* 7.170–38.530
“NETs-positive” *n* = 30	2.5365 0.894–6.554	3.1080 0.672–6.659	13.8965^a^ 9.631–18.078	1.6540^a^ 0.878–2.917	89.9980*^a^ 58.318–116.458	390.5785*^a^ 101.512–562.993	39.7150* 10.180–47.760
Control group *n* = 10	1.8775 0.193–2.886	2.1580 0.467–3.470	12.2155 10.698–14.881	0.9915 0.598–1.679	45.6460 31.010–68.515	291.4540 203.672–354.124	3.5200 1.330–7.070

^*^Statistically significant difference from the control group

^a^Statistically significant difference between “NETs-negative” and “NETs-positive” women

### NOX1 and NCF2 Concentration Assessment

The assessment of NOX1 and NCF2 concentrations in the serum of the tested women revealed that the mean concentrations of these proteins were the highest in “NETs-positive” patients, and that the concentrations in this group were significantly higher compared to the “NETs-negative” group.

No statistically significant differences in the concentrations of NOX1 and NCF2 were found between “NETs-positive” women and the control group. However, the levels of these proteins in the “NETs-negative” group were significantly lower compared to those in the control group (Table 2).

## MCP-1 and TNF-α Concentration Assessment

The assessment of the levels of MCP-1 and TNF-α in the serum of the tested patients indicated that the mean concentrations of these proinflammatory proteins were the highest in the “NETs-positive” women, and that the concentrations in this group were statistically significantly higher compared to the “NETs-negative” and control group.

No statistically significant differences in the concentrations of MCP-1 and TNF-α were observed between the “NETs-negative” and control group (Table 2).

### Determination of BPA Quantity

The chromatographic analysis revealed that the mean BPA concentration was the highest in the serum of “NETs-positive” group, with the value being over eightfold higher than that determined for the control group. Furthermore, the concentration of BPA was statistically significantly higher in the serum of “NETs-negative” patients compared to that in the control group (Table 2).

### Correlation Analysis

**Fig. 3:**
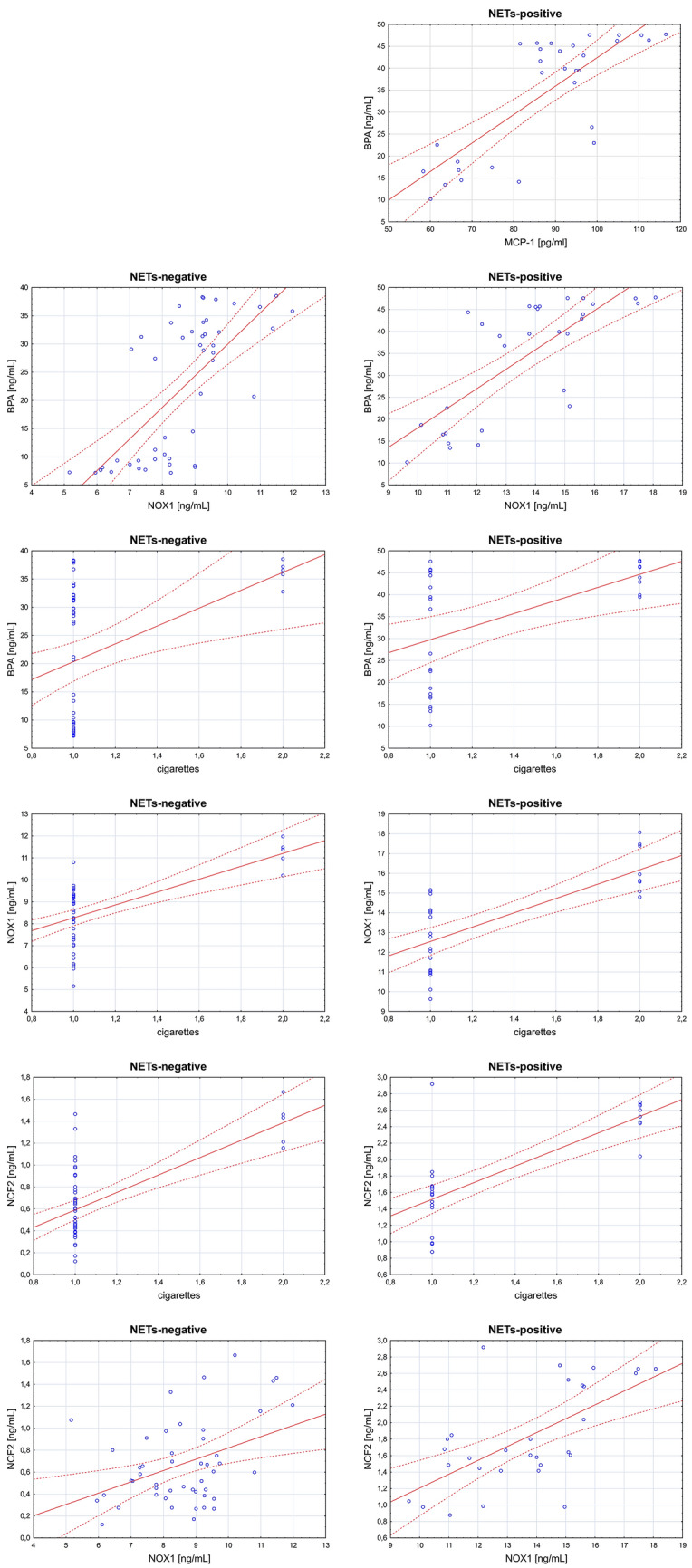
Correlations between the assessed parameters. Nonparametric Spearman’s rank correlation; 1.0—not smoking, 2.0—smoking

Figure 3 presents the statistically significant correlations observed between the parameters assessed in the study.

## Discussion

This study showed high concentrations of NADPH oxidase subunits in the serum of women with miscarriage who had high levels of NET biomarkers in their placental tissue, as observed in our previous study, which confirmed that NETs play a role in the premature termination of gestation (Omeljaniuk et al. 2020). Elevated concentrations of NOX1 and NCF2 in “NETs-positive” women with miscarriage seem to indicate the increased activity of NADPH oxidase, a key enzyme involved in the initiation of NET formation (Leung et al. 2021). The present study also showed a correlation between the levels of NOX1 and NCF2 in “NETs-positive” patients, which suggests that these proteins could potentially take part in the formation of NETs. The lack of statistical significance between the NOX1 and NCF2 results in the control group compared to the “NETs-positive” group is probably due to the number of study participants. The results were at the borderline of statistical significance, with a clear upward trend in “NET-positive” patients. Another reason for the lack of clear differences may be the ability of neutrophils to respond locally, to form NETs in the placenta, and to manifestless peripheral symptoms of NETosis. On the other hand, lower values of the tested proteins in the group of “NET-negative” patients indicate non-immunological causes of miscarriage.

Increased activation of oxidase leads to excessive generation of NETs through reactive oxygen species (ROS)/NET reaction cascade. High concentrations of NOX1 and NCF2 can disrupt oxidative–antioxidative balance, leading to the release of reactive oxygen and nitrogen species outside of the cell along with NETs. A study by Cui et al. (2006) demonstrated that overexpression of NOX1 correlated with elevated oxidative stress in the placenta of women who had preeclampsia. In turn, Troiano et al. (2016) observed that enhanced ROS production involving NOX oxidases resulted in endothelial dysfunction in pregnant rats with hypertension. Gomes et al. (2012) found that activated trophoblast cells produced ROS involving NADPH oxidase in mice. As there are no studies focusing on NOX1 and NCF2 in pregnant women or women with miscarriage in the literature, we could not compare the results obtained in this study. However, an earlier study confirmed that oxidative–antioxidative balance was disturbed in women with miscarriage (Omeljaniuk et al. 2015). A healthy pregnancy is characterized by an equilibrium between the activity of pro-oxidative factors, such as free radicals, and the efficiency of the antioxidant system. Several elements including Zn, Se, and Mn are components of the enzymes that participate in the first phase of the free radical scavenging process. In addition, trace elements form the prosthetic groups of some of these enzymes. Selenium is found in the active center of glutathione peroxidise (GSH-Px) and is responsible for the inactivation of free radicals in cells. The elements Zn, Cu, and Mn present in Cu Zn superoxide dismutase and Mn superoxide dismutase play a key role in the conversion of superoxide anion radicals to less harmful H_2_O_2_ (dihydrogen dioxide) (Gałecka et al. 2008). Advanced and irreversible stage of miscarriage is marked by a significant increase in the systemic markers of oxidative stress, but this change cannot be found during a mild disturbance in a healthy pregnancy. Oxidative stress within the placenta causes disturbances in fetal development (Al-Kunani et al. 2001; Gupta et al. 2007; Paszkowski and Łagód 2001). In our previous study, we showed that the total antioxidant status and serum Cu concentration were significantly lower in women who had miscarriage in the first trimester of pregnancy, while Mn level was higher compared to women with a normal pregnancy. The levels of Se, Cu, and Mn were found to be significantly higher in the tissue of patients after miscarriage, whereas the level of Zn was lower compared to pregnant women who had normal birth (Omeljaniuk et al. 2015).

In the present study, the extracellular presence of NETs was accompanied by inflammation in “NETs-positive” women, which may be related to the high serum levels of MCP-1 and TNF-α. In a study on women in the first trimester of pregnancy, Matta et al. (2007) observed that the expression of MCP-1 protein was regulated by thrombin in the decidua cells. In another study on a group of women with preeclampsia, Lockwood et al. (2006) found that TNF-α enhanced the chemotactic effect of this protein in the decidua cells during the early phase of the first trimester. Excessive macrophage recruitment can result in impaired trophoblast invasion, which is a primary placental defect associated with preeclampsia (Lockwood et al. 2007). Chaiworapongsa et al. (2002) observed elevated MCP-1 concentrations in women who had miscarriage due to amniocentesis during the first trimester, which highlights that the loss of pregnancy may have a direct relationship with the procedure performed. However, the results of these authors are inconsistent with those of Whitcomb et al. (2007), who did not observe any relationship between the risk of miscarriage and MCP-1 levels in the serum samples collected from patients at least ten days prior to miscarriage. The high level of MCP-1 in the serum collected from patients immediately after miscarriage and the lack of changes a few days before miscarriage suggest the involvement of this protein in the final stage of miscarriage, as indicated by the results of our study.

The high levels of BPA determined in the serum of women with miscarriage in the present study suggest that this xenoestrogen may play a role in the process of miscarriage. Similar to estrogens, BPA can interact with the endocrine system and modulate its functions by binding to membrane and/or nuclear estrogen receptors, often affecting human health, especially the reproductive process and fetal development (Kharrazian 2014; Lathi et al. 2014). Zbucka-Krętowska et al. (2018) described for the first time the inhibitory effect of BPA on fatty acid amide hydrolase, which may cause an increase in the level of endocannabinoids in pregnant women. Exposure to BPA and elevated level of endocannabinoids are some of the risk factors for miscarriage (Zbucka-Krętowska et al. 2018). Lathi et al. (2014) observed a significant correlation between the concentration of BPA and miscarriage in the first trimester of pregnancy. The positive correlation between exposure to BPA and the level of NOX1 in the serum of women with miscarriage shown by the present study suggests the existence of a relationship between high BPA concentration and increased activity of the NADPH oxidase complex, which may result in miscarriage through the activation of ROS/NETs pathway. The highest BPA concentration observed in “NETs-positive” women in this study suggests that BPA potentially influences the formation of NETs. These results support the findings of a previous study by Ratajczak-Wrona et al. (2021), who found an increase in NADPH oxidase and NETs after the exposure of neutrophils of healthy women to BPA. Moreover, the positive correlation between exposure to BPA and the level of MCP-1 protein can indicate the development of an inflammatory condition associated with the accumulation of this xenoestrogen. Liang et al. (2020) demonstrated that exposure to BPA may cause oxidative stress and disrupt the immune balance in women with relapsing miscarriage. Zheng et al. (2012) analyzed the relationships between BPA concentration, passive cigarette smoking, and the number of miscarriages in a group of women and observed higher BPA concentrations in women with miscarriage, who passively smoked tobacco products. The authors pointed out that the concentration of BPA increased in women with subsequent miscarriage events (Zheng et al. 2012). The present study also showed a strong positive correlation between BPA concentration and tobacco smoking in women with miscarriage, which suggests that BPA increases the risk of pregnancy loss by activating the ROS/NETs pathway due to its concomitant correlation with NOX1 and NCF2.

## Conclusions

The present study showed that BPA could be possibly involved in the course of miscarriage through the formation of NETs. The results obtained in this study with our previous observations are graphically illustrated in Fig. 1. Our conclusions are consistent with those of other authors and indicate the need to limit the exposure of humans to BPA. This can be achieved by, for example, minimizing the consumption of canned food, reducing the use of plastic products, and avoiding heating of food in plastic containers (Mikołajewska et al. 2015; Pergialiotis et al. 2018). The results of our study suggest that immune biomarkers can be potential predictors of NET formation, as well as inflammation, in the assessment of the risk of miscarriage. However, estimating the level of hazardous BPA is challenging due to the nonlinear systemic response to this xenoestrogen and patient-to-patient variations. Overall, our study indicates that BPA exposure should be limited in women who are undergoing treatment for infertility or those with relapsing miscarriages (Xu et al. 2017).

## Supplementary Information

Below is the link to the electronic supplementary material.

Supplementary file1 (DOC 320 KB)
