# Supplementary material for: Bisphenol A: Potential Factor of Miscarriage in Women in the Context of the Phenomenon of Neutrophil Extracellular Traps
Source: Arch Immunol Ther Exp (Warsz). 2022 Oct 1;70(1):24. doi: 10.1007/s00005-022-00661-w (PMC9526682; doi:10.1007/s00005-022-00661-w)
Supplement: Supplementary file 1 — Supplementary file1 (DOC 320 KB) [file 5_2022_661_MOESM1_ESM.doc]

**Supplement 1.** All data – statistic

| **Parameters** | **I group**  **Women “NETs-negative”** *n*=48 | | | | | | | | | | | |
| --- | --- | --- | --- | --- | --- | --- | --- | --- | --- | --- | --- | --- |
|  | mean | median | minimum | maximum | lower quartile  (Q1) | upper quartile  (Q3) | variance | SD | SE | skewness | se.skew | kurtosis |
| **Anty-PR3** | 1.4188 | 1.2720 | 0.5090 | 4.1370 | 0.9640 | 1.7685 | 0.47 | 0.6848 | 0.0988 | 1.6416 | 0.3431 | 4.1339 |
| **Anty-MPO** | 1.5472 | 1.3130 | 0.1960 | 8.0360 | 0.9135 | 1.6125 | 2.05 | 1.4329 | 0.2068 | 3.6071 | 0.3431 | 14.4044 |
| **NOX1** | 8.5752 | 8.7630 | 5.1570 | 11.9790 | 7.6260 | 9.3230 | 2.21 | 1.4873 | 0.2146 | 0.0194 | 0.3431 | 0.0250 |
| **NCF2** | 0.6735 | 0.5900 | 0.1220 | 1.6660 | 0.3925 | 0.9085 | 0.14 | 0.3788 | 0.0546 | 0.9257 | 0.3431 | 0.1153 |
| **MCP-1** | 58.5976 | 59.4060 | 33.1730 | 80.2240 | 47.6200 | 68.4770 | 175.85 | 13.2610 | 1.9140 | –0.1539 | 0.3431 | –0.8920 |
| **TNF-α** | 299.4987 | 307.5325 | 135.3230 | 553.3920 | 186.5080 | 388.0720 | 13756.43 | 117.2878 | 16.9290 | 0.2078 | 0.3431 | –1.0964 |
| **BPA** | 21.9829 | 27.2700 | 7.1700 | 38.5300 | 8.6450 | 32.4800 | 148.00 | 12.1657 | 1.7559 | –0.0632 | 0.3431 | –1.7968 |

| **Parameters** | **II group**  **Women “NETs-positive”** *n*=30 | | | | | | | | | | | |
| --- | --- | --- | --- | --- | --- | --- | --- | --- | --- | --- | --- | --- |
|  | mean | median | minimum | maximum | lower quartile  (Q1) | upper quartile  (Q3) | variance | SD | SE | skewness | se.skew | kurtosis |
| **Anty-PR3** | 2.7612 | 2.5365 | 0.8940 | 6.5540 | 1.8130 | 3.3220 | 1.76 | 1.3256 | 0.2420 | 1.2100 | 0.4268 | 1.8576 |
| **Anty-MPO** | 3.1612 | 3.1080 | 0.6720 | 6.6590 | 1.6770 | 4.3290 | 2.67 | 1.6340 | 0.2983 | 0.2865 | 0.4268 | –0.5158 |
| **NOX1** | 13.6355 | 13.8965 | 9.6310 | 18.0780 | 11.7050 | 15.1570 | 5.22 | 2.2845 | 0.4171 | 0.0828 | 0.4268 | –0.8580 |
| **NCF2** | 1.8190 | 1.6540 | 0.8780 | 2.9170 | 1.4480 | 2.4540 | 0.37 | 0.6049 | 0.1104 | 0.2891 | 0.4268 | –1.0474 |
| **MCP-1** | 87.3604 | 89.9980 | 58.3180 | 116.4580 | 74.7880 | 98.1610 | 264.84 | 16.2740 | 2.9712 | –0.2786 | 0.4268 | –0.7708 |
| **TNF-α** | 380.6815 | 390.5785 | 101.5120 | 562.9930 | 307.6570 | 462.1780 | 13661.98 | 116.8845 | 21.3400 | –0.6693 | 0.4268 | 0.0568 |
| **BPA** | 34.2220 | 39.7150 | 10.1800 | 47.7600 | 18.7100 | 45.7100 | 180.50 | 13.4350 | 2.4528 | –0.5910 | 0.4268 | –1.4230 |

| **Parameters** | **Control group** *n*=10 | | | | | | | | | | | |
| --- | --- | --- | --- | --- | --- | --- | --- | --- | --- | --- | --- | --- |
|  | mean | median | minimum | maximum | lower quartile  (Q1) | upper quartile  (Q3) | variance | SD | SE | skewness | se.skew | kurtosis |
| **Anty-PR3** | 1.7199 | 1.8775 | 0.1930 | 2.8860 | 1.2550 | 2.2880 | 0.81 | 0.9021 | 0.2852 | –0.6068 | 0.6870 | –0.5867 |
| **Anty-MPO** | 2.0185 | 2.1580 | 0.4670 | 3.4700 | 0.8560 | 3.1590 | 1.32 | 1.1483 | 0.3631 | –0.1931 | 0.6870 | –1.6925 |
| **NOX1** | 12.5104 | 12.2155 | 10.6980 | 14.8810 | 11.0910 | 14.0040 | 2.26 | 1.5020 | 0.4749 | 0.4666 | 0.6870 | –1.2362 |
| **NCF2** | 1.0976 | 0.9915 | 0.5980 | 1.6790 | 0.8780 | 1.5700 | 0.15 | 0.3856 | 0.1219 | 0.5322 | 0.6870 | –1.1143 |
| **MCP-1** | 48.5290 | 45.6460 | 31.0100 | 68.5150 | 36.5800 | 64.9060 | 198.38 | 14.0846 | 4.4539 | 0.3457 | 0.6870 | –1.4142 |
| **TNF-α** | 284.8664 | 291.4540 | 203.6720 | 354.1240 | 255.2180 | 323.3180 | 2191.55 | 46.8140 | 14.8039 | –0.4417 | 0.6870 | –0.4028 |
| **BPA** | 4.0060 | 3.5200 | 1.3300 | 7.0700 | 2.6600 | 5.4500 | 3.78 | 1.9430 | 0.6144 | 0.3502 | 0.6870 | –0.9646 |
